# Energy Transfer Study on Tb^3+^/Eu^3+^ Co-Activated Sol-Gel Glass-Ceramic Materials Containing MF_3_ (M = Y, La) Nanocrystals for NUV Optoelectronic Devices

**DOI:** 10.3390/ma13112522

**Published:** 2020-06-01

**Authors:** Natalia Pawlik, Barbara Szpikowska-Sroka, Wojciech A. Pisarski

**Affiliations:** Institute of Chemistry, University of Silesia, 40-007 Katowice, Poland; barbara.szpikowska-sroka@us.edu.pl

**Keywords:** Tb^3+^/Eu^3+^ energy transfer, sol-gel technique, glass-ceramics, NUV excitation

## Abstract

In the present work, the Tb^3+^/Eu^3+^ co-activated sol-gel glass-ceramic materials (GCs) containing MF_3_ (M = Y, La) nanocrystals were fabricated during controlled heat-treatment of silicate xerogels at 350 °C. The studies of Tb^3+^ → Eu^3+^ energy transfer process (ET) were performed by excitation and emission spectra along with luminescence decay analysis. The co-activated xerogels and GCs exhibit multicolor emission originated from 4f^n^–4f^n^ optical transitions of Tb^3+^ (^5^D_4_ → ^7^F_J_, J = 6–3) as well as Eu^3+^ ions (^5^D_0_ → ^7^F_J_, J = 0–4). Based on recorded decay curves, it was found that there is a significant prolongation in luminescence lifetimes of the ^5^D_4_ (Tb^3+^) and the ^5^D_0_ (Eu^3+^) levels after the controlled heat-treatment of xerogels. Moreover, for both types of prepared GCs, an increase in ET efficiency was also observed (from η_ET_ ≈ 16% for xerogels up to η_ET_ = 37.3% for SiO_2_-YF_3_ GCs and η_ET_ = 60.8% for SiO_2_-LaF_3_ GCs). The changes in photoluminescence behavior of rare-earth (RE^3+^) dopants clearly evidenced their partial segregation inside low-phonon energy fluoride environment. The obtained results suggest that prepared SiO_2_-MF_3_:Tb^3+^, Eu^3+^ GC materials could be considered for use as optical elements in RGB-lighting optoelectronic devices operating under near-ultraviolet (NUV) excitation.

## 1. Introduction

The development of materials dedicated to photonic applications is a frontier area of current materials engineering research [1,2]. Thus, considerable efforts are still being made to improve the optical parameters of such materials, e.g., the tuning of emissions in the desired spectrum range. In this case, the willingly studied pathway for generation a white light is related to the mixing of three primary colors—red, green and blue (RGB)—in optical materials. Such red-green-blue multicolor visible light can be achieved via up-conversion of near-infrared radiation (NIR) [3,4,5,6,7] or via the conversion of near-ultraviolet (NUV) photons [8,9,10]. Since rare-earth ions (RE^3+^) exhibit a broad range of emission in the visible (VIS) spectral scope via interactions with NIR and NUV irradiation, they are considered as essential parts in the development of white-light-emitting RGB materials [11,12]. The first of abovementioned ways of generating RGB emission can be realized via NIR up-conversion excitation in doubly (e.g., Yb^3+^/Er^3+^ [3,4], Yb^3+^/Tm^3+^ [4]) and triply doped (e.g., Yb^3+^/Er^3+^/Tm^3+^ [5,6], Tb^3+^/Tm^3+^/Yb^3+^ [7]) optical systems. For example, for Yb^3+^/Er^3+^/Tm^3+^ triply doped β-NaYF_4_ microrods, Er^3+^ ions are responsible for the generation of red (the ^4^F_9/2_ → ^4^I_15/2_ transition) as well as green (the ^2^H_11/2_ → ^4^I_15/2_ and the ^4^S_3/2_ → ^4^I_15/2_ transitions) emissions through a two-photon absorption process involved in Yb^3+^ → Er^3+^ energy transfer. Simultaneously, due to the three-photon assisted process of Yb^3+^ → Tm^3+^ energy transfer, Tm^3+^ ions are able to emit blue light (the ^1^D_2_ → ^3^F_4_ and the ^1^G_4_ → ^3^H_6_ transitions) [5]. The second of the abovementioned routes can be realized via the successful conversion of NUV irradiation into VIS light. In this matter, Eu^3+^ ions are treated as a red or reddish-orange light source (the ^5^D_0_ → ^7^F_J_ transitions, J = 0–4) and Tb^3+^ ions are considered as one of the most important sources of green emission (the ^5^D_4_ → ^7^F_5_ transition). Therefore, co-doping with Tb^3+^/Eu^3+^ ions seems to be a promising strategy for the generation of multicolor luminescence, which plays a key role in RGB optical materials [13,14,15,16].

Another particularly important point in the field of substantial enhancement, the luminescence of RE^3+^ ions is related to the selection of a suitable host lattice with low phonon energy. Since the phonon energies of fluorides—usually in a range from 400 up to 500 cm^−1^ [17]—are significantly lower compared to phosphates (~1250 cm^−1^) [18] or borates (~1350 cm^−1^) [19], they are considered as great candidates for generating an efficient and long-lived luminescence of RE^3+^. Among fluorides, special attention should be paid to YF_3_ and LaF_3_ crystal phases characterized by wide band gap (>10 eV) and exceptionally low-phonon energies equal to ~358 as well as ~350 cm^−1^, respectively, in which M^3+^ (M = Y, La) cations from crystal lattices can be easily substituted by RE^3+^ ions without any charge compensation [20,21,22,23]. Due to the above reasons, the oxyfluoride glass-ceramic materials (GCs) containing fluoride nanocrystals are considered as an interesting class of advanced optical materials, which are frequently reported in the literature [24,25,26,27,28]. Indeed, they successfully combine the advantages of the individual fluoride crystal phase with good mechanical strength and thermal durability of oxide hosts [29,30]. It should also be noted that due to the compliance with the principles of green chemistry, the systematic elimination of PbF_2_ (characterized by exceptionally low-phonon energy equals to ~250 cm^−1^ [31]) during preparation is currently a very important aspect. Thus, taking the above considerations into account, Tb^3+^/Eu^3+^ co-doped oxyfluoride GCs containing selected fluoride phases (e.g., YF_3_ and LaF_3_) seems to be a good choice for the generation of efficient visible emissions.

The conventional melt-quenching method followed by controlled heat-treatment at specified time and temperature conditions is currently the most widely used technique for fabricating the class of oxyfluoride GCs [32,33,34,35,36]. On the other hand, the high melting temperatures of glass-forming components (e.g., 1450 °C, 1500 °C [37,38,39]) increase the risk of volatilization of the fluoride compounds, which may adversely affect the crystallization process of the fluoride fraction. Therefore, an alternative route to obtain oxyfluoride GCs is the sol-gel technique, characterized by low-temperature processing [40,41,42]. This method is based on hydrolysis, condensation and polycondensation reactions of organometallic precursors, usually alkoxysilanes Si(OR)_4_ (R = –CH_3_, –C_2_H_5_, etc.) in a liquid phase at room temperature [43]. The in-situ crystallization of fluoride phases is possible due to introduction of a fluorinating reagent into reaction system at initial stages, whose role is commonly played by trifluoroacetic acid (TFA) [44]. Since the sintering of sol-gel materials is carried out at significantly lower temperatures (usually <500 °C) than the conventional melt-quenching of glasses, a risk of volatilization of fluorides is adequately lower, meaning that sol-gel processing is preferred. Lower energy consumption also makes the sol-gel technique particularly advantageous and attractive from an environmental friendliness point of view. Simultaneously, to the best of our knowledge, the investigation of Tb^3+^/Eu^3+^ energy transfer process in oxyfluoride GCs is extremely rarely described in the available literature. In the literature, there is only one excellent description concentrated on YF_3_:Tb^3+^, Eu^3+^ nanocrystalline-based GCs fabricated from 44SiO_2_-28Al_2_O_3_-17NaF-(10 − x)YF_3_-TbF_3−x_EuF_3_ (x = 0, 0.1, 0.25, 1) glasses during their heat-treatment at 670 °C per 2 h, as far as we know [45]. Due to the above reasons, it seems to be justified to study the Tb^3+^ → Eu^3+^ energy transfer in sol-gel GCs containing MF_3_ (M = La or Y) nanocrystals.

In the present work, the sol-gel oxyfluoride GCs materials containing MF_3_:Tb^3+^, Eu^3+^ (M = Y, La) nanocrystals were successfully fabricated during controlled heat-treatment at low temperature (350 °C per 10 h) and characterized by detailed luminescence measurements. The studies were performed by means of excitation and emission spectra along with lifetime measurements. Based on photoluminescence results, the interactions between Tb^3+^ and Eu^3+^ dopant ions were systematically investigated and the incorporation of dopant ions into the fluoride environment was also analyzed.

## 2. Materials and Methods 

**Figure 1:**
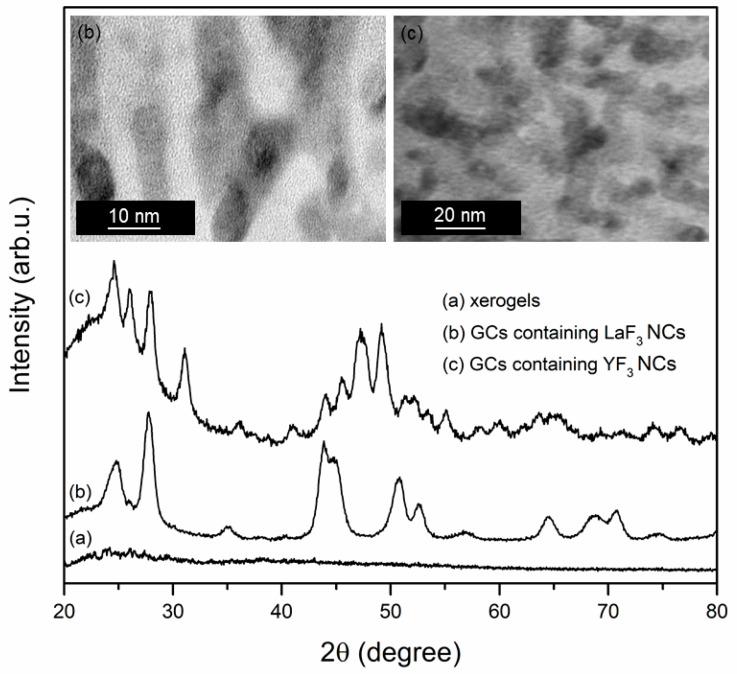
Transmission electron microscope (TEM) images and X-ray diffraction (XRD) patterns of fabricated xerogels and glass-ceramic samples.

The xerogels co-doped with Tb^3+^ and Eu^3+^ ions were synthesized using the low-temperature sol-gel method. All reagents used during the described procedure were taken from Aldrich Chemical Co. The sol-gel synthesis was started with the introduction of tetraethoxysilane (TEOS, 98%), ethyl alcohol (98%), deionized water (from Elix 3 system, Millipore, Molsheim, France) and acetic acid (99.5–99.9%) into round-bottom flasks. The molar ratio of components was equal to TEOS:C_2_H_5_OH:H_2_O:CH_3_COOH = 1:4:10:0.5 (90 wt.%). To perform hydrolysis and to initialize a condensation reaction, the components were stirred for 30 min. During the next step, the appropriate amounts of acetates, i.e., M(CH_3_COO)_3_ (M = Y, La; 99.9%) as well as Tb(CH_3_COO)_3_ (99.999%) and Eu(CH_3_COO)_3_ (99.999%) were weighed and dissolved in trifluoroacetic acid (TFA, 99%) and obtained mixtures were added into TEOS-based solutions. The molar ratio was equal to CF_3_COOH:M(CH_3_COO)_3_:Tb(CH_3_COO)_3_:Eu(CH_3_COO)_3_ = 5:1:0.05:0.05 (10 wt.%) (M = Y, La). The resultant solutions were mixed for another 60 min. After sol-gel synthesis, the obtained liquid sols were dried at 35 °C for 7 weeks to form colorless and transparent solid xerogels (denoted in the text as XGs). Their further transformation into glass-ceramic materials containing YF_3_ and LaF_3_ nanocrystals was realized by controlled heat-treatment in a muffle furnace (FCF 5 5SHP produced by Czylok, Jastrzębie-Zdrój, Poland) at 350 °C per 10 h (the temperature was raised by 10 °C/min from room temperature). After this procedure, the samples were slowly cooled down to room temperature (denoted in the text as SiO_2_-MF_3_, M = Y, La). The successful formation of fluoride nanocrystals (LaF_3_ NCs: *P6_3_/mmc*, ICDD PDF-2 No. 08-0461; YF_3_ NCs: *Pnma*, ICDD PDF-2 No. 32-1431) was verified using X-ray diffraction (XRD, X’Pert Pro diffractometer, Panalytical, Almelo, The Netherlands) and the nanocrystals imaging was done via high-resolution transmission electron microscope (HR-TEM, JEOL JEM 3010, Tokyo, Japan). The results are shown in Figure 1. The average diameters of fabricated nanocrystals were estimated to 8.1 nm for LaF_3_ and 15.4 nm for YF_3_. The in-situ formation of LaF_3_ and YF_3_ nanocrystals during the thermal decomposition of La(CF_3_COO)_3_ as well as Y(CF_3_COO)_3_ in applied heat-treatment conditions (350 °C, 10 h) was confirmed and reported in details in our previous works [46,47].

The excitation and emission spectra as well as decay curves were recorded on Horiba Jobin Yvon FluoroMax-4 spectrofluorimeter (Horiba Jobin Yvon, Longjumeau, France) supplied with 150 W Xe lamp. The spectra were recorded with ± 0.1 nm resolution and the decay curves were recorded with ± 2 μs accuracy. All structural and luminescence measurements were carried out at room temperature.

## 3. Results and Discussion

### 3.1. Excitation Spectra of Fabricated Tb^3+^,Eu^3+^ Co-Doped Sol-Gel Materials 

**Figure 2:**
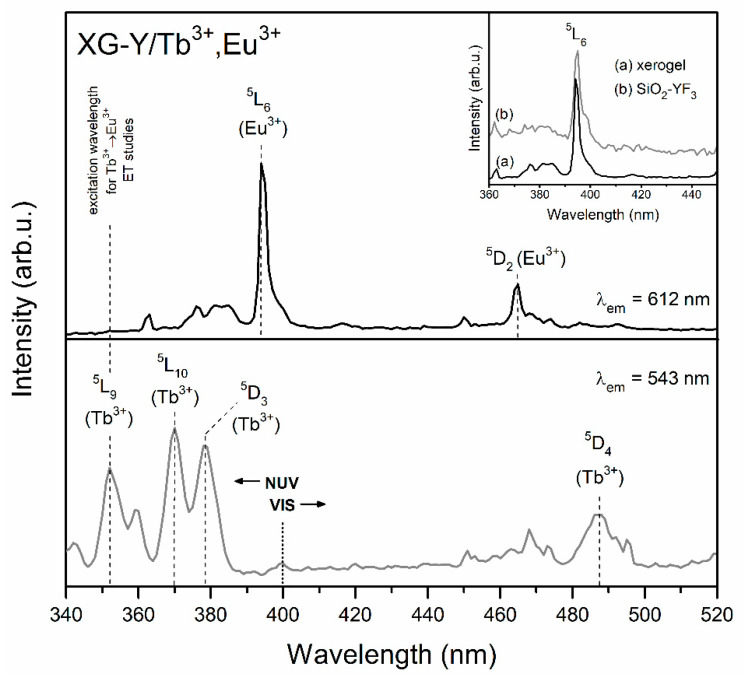
Photoluminescence excitation (PLE) spectra of XG-Y/Tb^3+^,Eu^3+^ co-doped sample, monitored at λ_em_ = 543 nm and λ_em_ = 612 nm.

**Figure 3:**
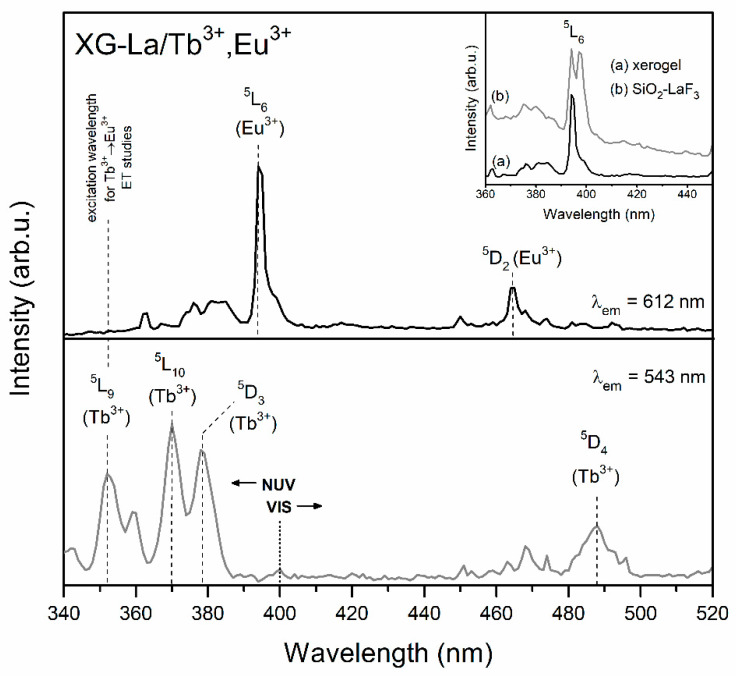
PLE spectra of XG-La/Tb^3+^,Eu^3+^ co-doped sample, monitored at λ_em_ = 543 nm and λ_em_ = 612 nm.

Figure 2 and Figure 3 present the photoluminescence excitation (PLE) spectra of fabricated Tb^3+^/Eu^3+^ co-doped xerogels. The PLE spectra were recorded in the spectral range from 340 to 520 nm and monitored at λ_em_ = 543 nm and λ_em_ = 612 nm emissions (the ^5^D_4_ → ^7^F_5_ green line of Tb^3+^ ions and the ^5^D_0_ → ^7^F_2_ red line of Eu^3+^, respectively). The recorded bands were attributed to the 4f^8^–4f^8^ and 4f^6^–4f^6^ intra-configurational transitions from both of optically active ions. The bands originating from Eu^3+^ were assigned to the following transitions: ^7^F_0_ → ^5^D_4_ (363 nm), ^7^F_0_ → ^5^G_J_, ^5^L_7_ (from 372 nm to 389 nm), ^7^F_0_ → ^5^L_6_ (394 nm) and ^7^F_0_ → ^5^D_2_ (464 nm). Among the group of excitation bands originated from Tb^3+^ ions, the transitions were ascribed to the electronic transitions from the ^7^F_6_ ground level into the subsequent upper-lying states: ^5^L_9_ (352 nm), ^5^L_10_ (370 nm), ^5^D_3_ (378 nm) and ^5^D_4_ (488 nm).

The selection of excitation parameters for further emission measurements and to study the Tb^3+^ → Eu^3+^ energy transfer process was done from the near-UV (NUV) irradiation area (<400 nm) due to the greater intensity of recorded excitation lines than in visible light (VIS) scope (>400 nm). Indeed, the optical elements considered to be used in RGB-lighting devices should operate under NUV excitation. Since the ^7^F_0_ → ^5^L_6_ excitation band of Eu^3+^ is the most intense, we decided to perform the luminescence measurements for Eu^3+^ ions using λ_exc_ = 394 nm wavelength.

As the band associated to the ^7^F_6_ → ^5^L_9_ transition of Tb^3+^ ions does not coincide with any excitation peak of Eu^3+^, the choice of λ_exc_ = 352 nm wavelength as the excitation source for the generation of the Tb^3+^ → Eu^3+^ energy transfer seems to be reasonable. Additionally, in order to compare the luminescence behavior of sol-gel samples co-doped with Tb^3+^/Eu^3+^ ions with samples singly doped with Tb^3+^ ions, all photoluminescence measurements for Tb^3+^ ions were performed using the λ_exc_ = 352 nm excitation line.

It should be also noted that for prepared GC samples, the ^7^F_0_ → ^5^L_6_ excitation line was split into two separated components, the maxima of which were located at 394 and 397 nm (shown in inset of Figure 2 and Figure 3). Similar results were reported by A.C. Yanes et al. [48] for sol-gel glass-ceramics with 89.9SiO_2_-10LaF_3_:0.1EuF_3_ (mol %) composition. Based on numerous photoluminescence measurements (excitation and emission spectra recorded at different temperatures: room temperature (RT), 100 and 13 K; analysis of emission spectra under excitation at 393 and 396 nm), it was clearly proven that the two PLE components are strictly related to the distribution of Eu^3+^ between the SiO_2_ sol-gel host (393 nm component) and LaF_3_ nanocrystals (396 nm component). Thus, in the case of our studied sol-gel samples, we also assumed that the origin of such a split after the controlled heat-treatment of xerogels (XG-Y/Tb^3+^,Eu^3+^ and XG-La/Tb^3+^,Eu^3+^) is related to the partial migration of rare-earths from silicate sol-gel host into MF_3_ (M = Y, La) nanocrystals. It is quite interesting that deeper splitting of the ^7^F_0_ → ^5^L_6_ band was observed for SiO_2_-LaF_3_:Tb^3+^,Eu^3+^ GC and, in consequence, two strong components with maxima at 394 and 397 nm are visible. For the SiO_2_-YF_3_:Tb^3+^,Eu^3+^ GC sample, the 397 nm component is visible as a weak shoulder. This effect may suggest more efficient segregation of dopant ions in LaF_3_ than YF_3_ nanocrystals. Analogous results were reported earlier by us for singly doped SiO_2_-YF_3_:Eu^3+^ [46] and SiO_2_-LaF_3_:Eu^3+^ [47] GC sol-gel systems.

### 3.2. Influence of Controlled Heat-Treatment at 350 °C on Tb^3+^ → Eu^3+^ Energy Transfer

**Figure 4:**
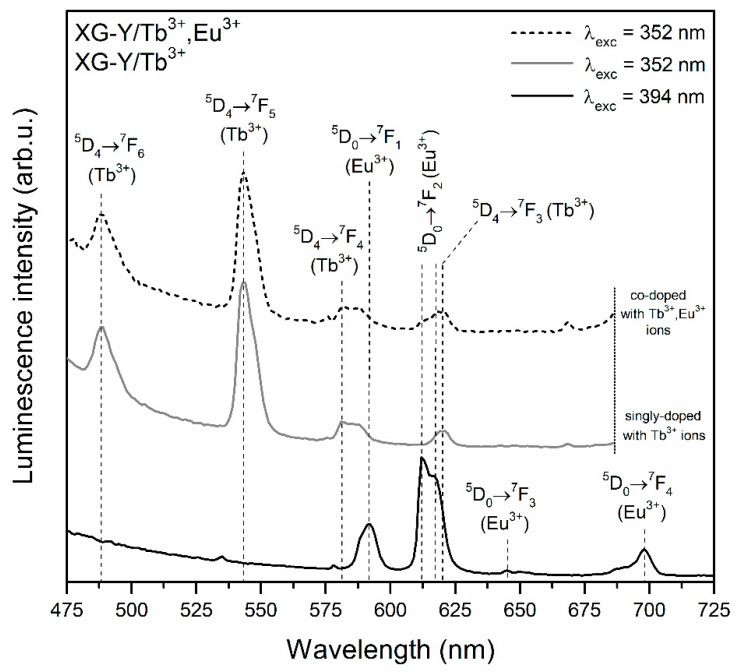
Photoluminescence (PL) spectra of XG-Y/Tb^3+^ and XG-Y/Tb^3+^,Eu^3+^ samples recorded upon near-ultraviolet (NUV) illumination at λ_exc_ = 352 nm and λ_exc_ = 394 nm.

**Figure 5:**
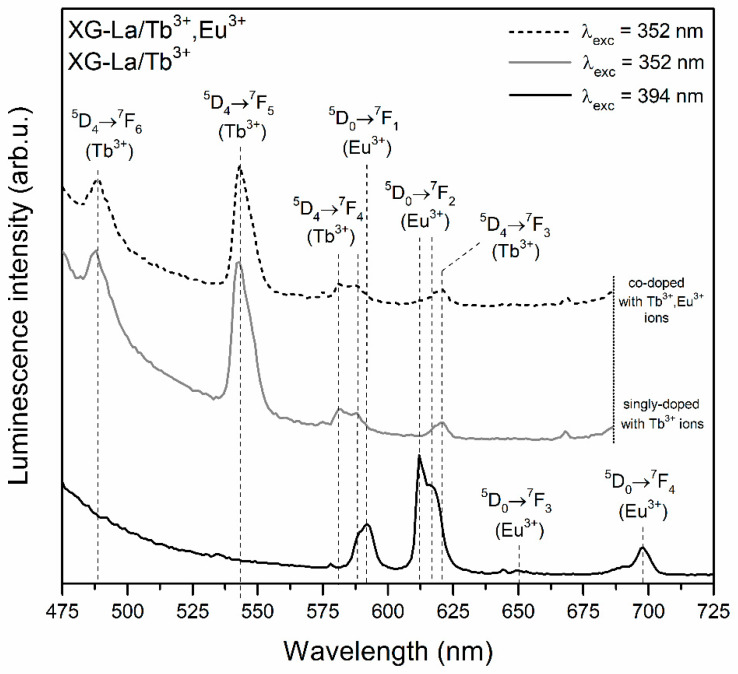
PL spectra of XG-La/Tb^3+^ and XG-La/Tb^3+^,Eu^3+^ samples recorded upon NUV illumination at λ_exc_ = 352 nm and λ_exc_ = 394 nm.

The photoluminescence (PL) spectra recorded for prepared silicate xerogels are illustrated in Figure 4 (XG-Y/Tb^3+^ and XG-Y/Tb^3+^,Eu^3+^) as well as in Figure 5 (XG-La/Tb^3+^ and XG-La/Tb^3+^,Eu^3+^). The excitation of Eu^3+^ ions using the λ_exc_ = 394 nm wavelength resulted in the appearance of luminescence bands located within the reddish-orange light area: ^5^D_0_ → ^7^F_0_ (578 nm), ^5^D_0_ → ^7^F_1_ (592 nm), ^5^D_0_ → ^7^F_2_ (611 nm), ^5^D_0_ → ^7^F_3_ (645 nm), and ^5^D_0_ → ^7^F_4_ (698 nm). Since the local framework around Eu^3+^ ions in sol-gel host is non-symmetric, the most intense emission line corresponds to the ^5^D_0_ → ^7^F_2_ electric-dipole transition and red-to-orange ratio (R/O) values for fabricated xerogels are relatively high (R/O = 3.01 for XG-Y/Tb^3+^,Eu^3+^; R/O = 2.78 for XG-La/Tb^3+^,Eu^3+^).

The PL spectra recorded upon excitation at λ_exc_ = 352 nm for xerogels singly doped with Tb^3+^ ions revealed two emission bands in the bluish-green spectral scope, i.e., ^5^D_4_ → ^7^F_6_ (488 nm) and the most prominent ^5^D_4_ → ^7^F_5_ (543 nm) line. Two another emission bands of Tb^3+^ ions were detected in the yellowish-red range: ^5^D_4_ → ^7^F_4_ (584 nm) and ^5^D_4_ → ^7^F_3_ (619 nm). The excitation of Tb^3+^/Eu^3+^ co-doped xerogels using λ_exc_ = 352 nm wavelength led to generating the characteristic emission lines of Tb^3+^ ions; however, it should be noted that some spectral broadening of the ^5^D_4_ → ^7^F_4_ as well as the ^5^D_4_ → ^7^F_3_ bands was observed. Such broadening is a consequence of the energy transfer process from Tb^3+^ to Eu^3+^, which resulted in the appearance of additional luminescence coming from Eu^3+^ dopant (^5^D_0_ → ^7^F_1_ and ^5^D_0_ → ^7^F_2_ transitions) [49,50,51,52]. In general, the spectral matching of donor’s emission (Tb^3+^) and acceptor’s excitation (Eu^3+^) regions is a fundamental condition for energy transfer occurrence. In this way, upon irradiation using λ_exc_ = 352 nm line from NUV spectral region, Tb^3+^ ions could be successfully pumped into the ^5^L_9_ level and then the non-radiative de-activation to the ^5^D_4_ state takes place. Since there is spectral overlapping between the ^5^D_4_ → ^7^F_5_,^7^F_4_ emissions of Tb^3+^and the ^7^F_1_ → ^5^D_1_ and the ^7^F_0_, ^7^F_1_ → ^5^D_0_ excitation bands of Eu^3+^, the energy could be successfully transferred from Tb^3+^ into Eu^3+^ ions as follows [53,54]:

^5^D_4_ (Tb^3+^) + ^7^F_1_ (Eu^3+^) → ^7^F_5_ (Tb^3+^) + ^5^D_1_ (Eu^3+^) (1),

^5^D_4_ (Tb^3+^) + (^7^F_0_, ^7^F_1_) (Eu^3+^) → ^7^F_4_ (Tb^3+^) + ^5^D_0_ (Eu^3+^) (2).

**Figure 6:**
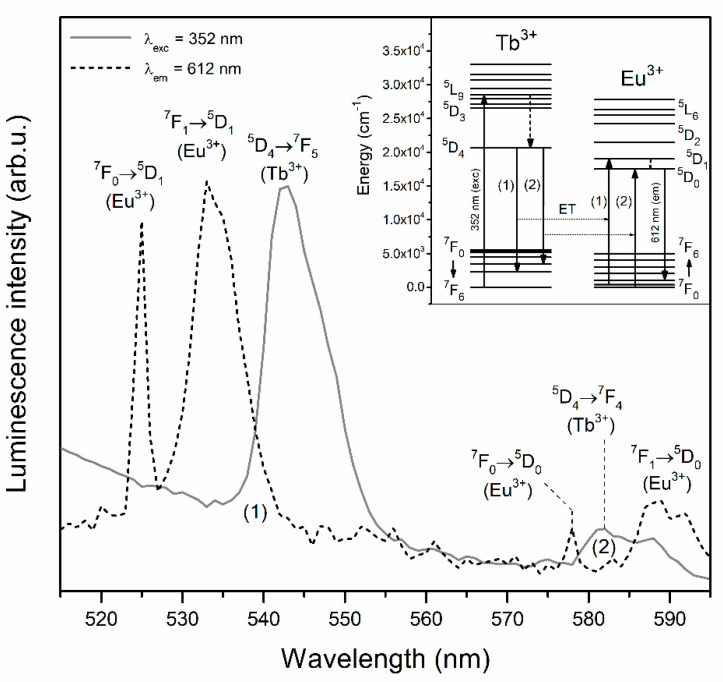
An overlap of Tb^3+^ emission (λ_exc_ = 352 nm) and Eu^3+^ excitation (λ_em_ = 612 nm) regions and illustration of energy levels involved in Tb^3+^ → Eu^3+^ energy transfer process.

Hence, among the characteristic emission lines from Tb^3+^ ions, additional bands originated from Eu^3+^ can also be recorded. The matching of Tb^3+^ emission and Eu^3+^ excitation as well as illustration of energy levels involved in Tb^3+^ → Eu^3+^ energy transfer process are depicted in Figure 6.

**Figure 7:**
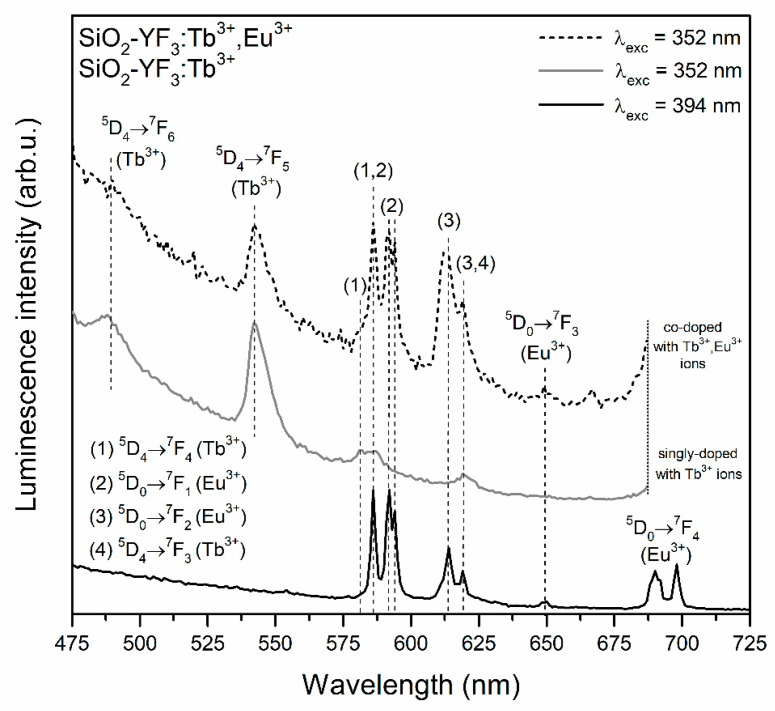
PL spectra of SiO_2_-YF_3_:Tb^3+^ and SiO_2_-YF_3_:Tb^3+^,Eu^3+^ samples recorded under NUV excitation at λ_exc_ = 352 nm and λ_exc_ = 394 nm.

**Figure 8:**
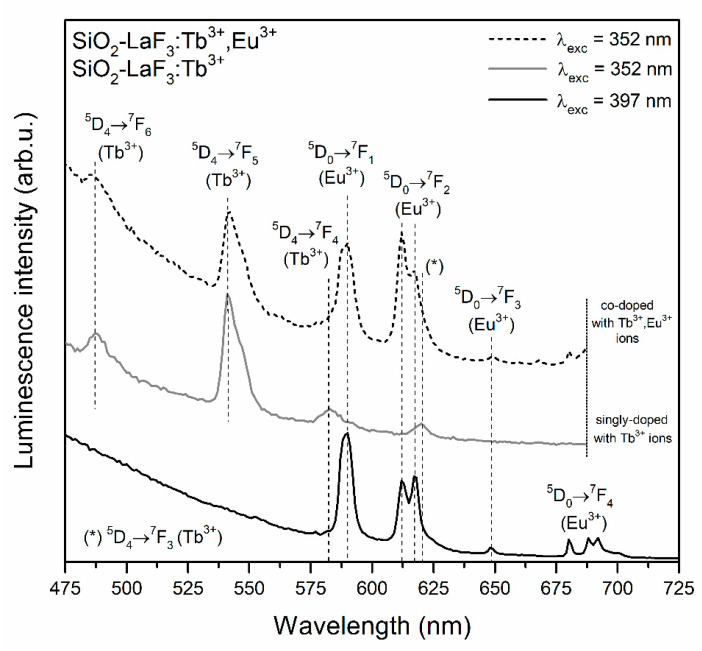
PL spectra of SiO_2_-LaF_3_:Tb^3+^ and SiO_2_-LaF_3_:Tb^3+^, Eu^3+^ samples recorded under NUV excitation at λ_exc_ = 352 nm and λ_exc_ = 397 nm.

The PL spectra recorded for glass-ceramic materials are shown in Figure 7 (SiO_2_-YF_3_:Tb^3+^ and SiO_2_-YF_3_:Tb^3+^,Eu^3+^) and Figure 8 (SiO_2_-LaF_3_:Tb^3+^ and SiO_2_-LaF_3_:Tb^3+^,Eu^3+^). For both types of prepared co-doped GC, a well-resolved Stark structure of recorded luminescence bands of Eu^3+^ ions was observed, which points to crystalline-like environment around them. For SiO_2_-YF_3_:Tb^3+^, Eu^3+^ GCs the following maxima are located at: 586/592/594 nm (^5^D_0_ → ^7^F_1_), 614 nm/619 nm (^5^D_0_ → ^7^F_2_), 650 nm (^5^D_0_ → ^7^F_3_) and 690/692/698 nm (^5^D_0_ → ^7^F_4_), while for SiO_2_-YF_3_:Tb^3+^, Eu^3+^ GCs, the maxima of individual emission bands were detected at the following wavelengths: 590 nm (^5^D_0_ → ^7^F_1_), 612 nm/618 nm (^5^D_0_ → ^7^F_2_), 649 nm (^5^D_0_ → ^7^F_3_) and 680/688/692 nm (^5^D_0_ → ^7^F_4_). Such clear splitting is a consequence of the partial segregation of Eu^3+^ ions inside fluoride crystal lattices. Indeed, when Eu^3+^ ions are inserted into the crystal lattice, the subsequent energy levels get split by the crystal-field effect and the number of sub-levels depends on the local site symmetry. It is reported in the literature that in YF_3_ and LaF_3_ crystal lattices, Eu^3+^ ions occupy C_s_ and C_2v_ point symmetry sites, respectively [55,56]. If Eu^3+^ ions occupy C_s_ and C_2v_ site symmetry, the J term of the ^7^F_J_ levels should split into three (J = 1), five (J = 2), seven (J = 3) and nine (J = 4) sub-levels [57]. However, observation of such strong splitting for glass-ceramic systems is quite difficult due to the partial distribution of Eu^3+^ ions within the amorphous sol-gel host. Moreover, compared to xerogels, a significant increase in intensity of the ^5^D_0_ → ^7^F_1_ magnetic-dipole transition band was observed, while the intensity of the ^5^D_0_ → ^7^F_2_ electric-dipole transition is strongly inhibited. Generally, the ^5^D_0_ → ^7^F_1_ magnetic-dipole transition is orbitally allowed and practically insensitive to symmetry in the local environment around Eu^3+^ ions. Conversely, the ^5^D_0_ → ^7^F_0,2–4_ electric-dipole transitions are forbidden in centrosymmetric sites due to the same parity of energy levels. However, if Eu^3+^ ions are located in non-centrosymmetric sites, the transitions became allowed as forced electric-dipole transitions due to the mixing of wavefunctions of 4f^6^ sublevels with different J values. Therefore, the ratio of integrated luminescence intensity of the ^5^D_0_ → ^7^F_2_ electric-dipole transition to the ^5^D_0_ → ^7^F_1_ magnetic-dipole transition can inform us about local symmetry around Eu^3+^ ions and is called the R/O ratio. A decrease in R/O-ratio value clearly suggests that local symmetry around Eu^3+^ ions is closer to an inversion center. The calculated R/O ratio values after controlled heat-treatment are relatively low (R/O = 0.42 for SiO_2_-YF_3_:Tb^3+^,Eu^3+^; R/O = 0.89 for SiO_2_-LaF_3_:Tb^3+^,Eu^3+^), which clearly points to the partial migration of Eu^3+^ ions from sol-gel host into more symmetric fluoride crystal phases.

For the prepared SiO_2_-MF_3_:Tb^3+^ GC samples, the characteristic emission bands corresponding to the transitions from the ^5^D_4_ excited level into the ^7^F_6_, ^7^F_5_, ^7^F_4_ and ^7^F_3_ lower-lying states were detected. In the case of SiO_2_-MF_3_:Tb^3+^,Eu^3+^ co-doped GCs, the mutual coincidence of the luminescence lines originating from both of the rare-earths was clearly observed after excitation at the λ_exc_ = 352 nm line. Therefore, an appearance of characteristic emission bands coming from Eu^3+^ ions upon excitation of Tb^3+^ confirms the occurrence of Tb^3+^ → Eu^3+^ energy transfer. 

It should be particularly pointed out that the intensity of Tb^3+^ emission bands decreased, which was accompanied by the simultaneous enhancement of Eu^3+^ luminescence. Such an effect is much more observable for GC samples than for precursor xerogels. Hence, it could suggest that both of the optically active dopants were incorporated into the crystal lattices of the YF_3_ as well as LaF_3_ phases, causing there to be a shorter distance between neighboring Tb^3+^-Eu^3+^ pairs in comparison with the sol-gel host. Thus, the Tb^3+^ → Eu^3+^ seems to be more efficient in glass-ceramic materials, which results in a much more intense emission of Eu^3+^ ions.

### 3.3. Luminescence Decay Analysis of the ^5^D_4_ (Tb^3+^) and the ^5^D_0_ (Eu^3+^) Levels in Sol-Gel Materials

To further optical examination of prepared xerogels and GC materials, the luminescence decay analysis of the ^5^D_4_ (Tb^3+^) and the ^5^D_0_ (Eu^3+^) excited states was performed. The analysis of decay profiles allows for the deeper characterization of the Tb^3+^ → Eu^3+^ energy transfer process and for establishing the relation between luminescence lifetimes and distribution of rare-earths within sol-gel materials.

**Figure 9:**
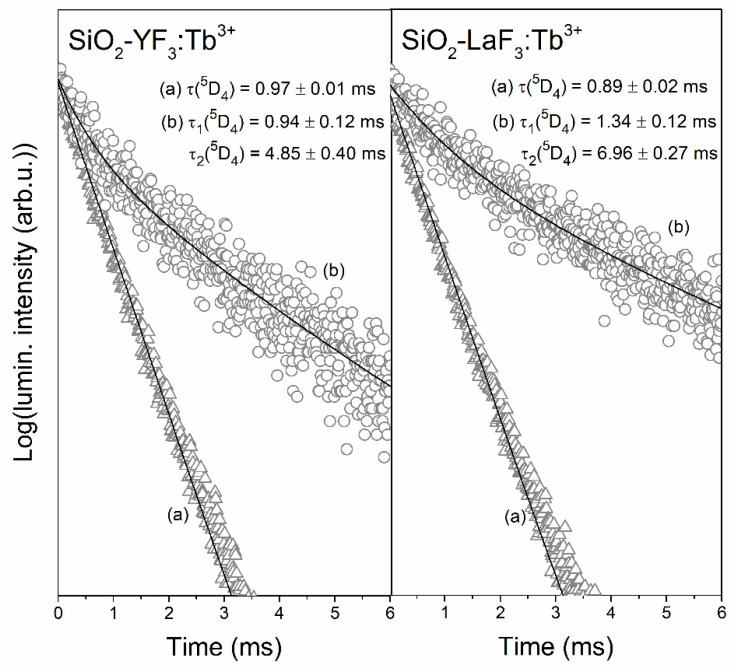
Luminescence decay curves of the ^5^D_4_ state of Tb^3+^ recorded for xerogels (**a**) and glass-ceramics (**b**) singly doped with Tb^3+^ ions.

**Figure 10:**
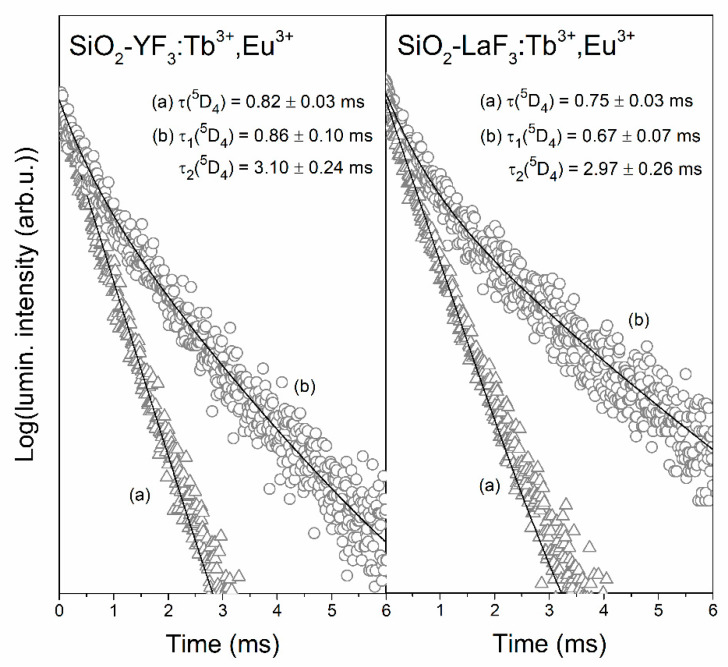
Luminescence decay curves of the ^5^D_4_ state of Tb^3+^ recorded for xerogels (**a**) and glass-ceramics (**b**) co-doped with Tb^3+^/Eu^3+^ ions.

Firstly, we compared the luminescence lifetimes of the ^5^D_4_ level of Tb^3+^ ions in singly doped sol-gel materials (Figure 9) and materials co-doped with Tb^3+^/Eu^3+^ ions (Figure 10). The decay curves were recorded upon NUV excitation (λ_exc_ = 352 nm) and monitoring a green emission related to the ^5^D_4_ → ^7^F_5_ transition of Tb^3+^ (λ_em_ = 543 nm). For xerogels, the curves are well-fitted to mono-exponential functions given by:

(1) $$I\left(t\right)/I_{0}=A\times \exp\left(-t/\tau \right),$$

where τ corresponds to the luminescence decay time. It was observed that the τ (^5^D_4_) estimated for XG-Y/Tb^3+^ and XG-La/Tb^3+^ is quite comparable and equals 0.97 ± 0.01 and 0.89 ± 0.02 ms, respectively. Such negligible differences in luminescence lifetime values are caused by considerable similarity of chemical environment around Tb^3+^ in fabricated silicate xerogels. Indeed, the non-radiative relaxation between subsequent J states might occur due to the interaction of electronic levels of RE^3+^ ions with suitable vibrational modes in their nearest surrounding. As was proven earlier by infrared measurements, some liquids (residual solvents used during sol-gel process and products of homo- and heterocondensation reactions) are retained in xerogel pores [46]. In this way, Tb^3+^ and Eu^3+^ ions are coordinated by CF_3_COO^−^ anions (C=O groups: ~1650 cm^−1^, C–F bond: ~1200 cm^−1^) and OH groups (>3000 cm^−1^). According to energy gap law, an increase in the non-radiative decay rate is assisted by the decreasing number of phonons needed to cover the energy gap, ∆E. Since OH groups are characterized by higher vibrational energy than CF_3_COO^−^ anions, we assumed that OH groups with higher phonon energies play a major role in non-radiative relaxation from the ^5^D_4_ state. In this way, a maximum of five OH phonons are needed to cover the ^5^D_4_ → ^7^F_0_ energy gap of Tb^3+^ ions (∆E = 15,000 cm^−1^); therefore, the probability of non-radiative depopulation from the ^5^D_4_ state in xerogels is relatively high.

A comparison of luminescence lifetimes of the ^5^D_4_ state for xerogels singly doped with Tb^3+^ ions and co-doped with Tb^3+^/Eu^3+^ ions reveals a slight shortening when Tb^3+^ ions coexist with Eu^3+^ ions in the same host. Indeed, it was reported that there was a decrease from 0.97 ± 0.01 to 0.82 ± 0.03 ms for XG-Y/Tb^3+^,Eu^3+^ and from 0.89 ± 0.02 to 0.75 ± 0.03 ms for XG-La/Tb^3+^,Eu^3^. Therefore, such an effect is a clear evidence of Tb^3+^ → Eu^3+^ energy transfer occurrence. The energy transfer efficiency (η_ET_) between Tb^3+^ and Eu^3+^ ions could be expressed by the following Equation [54,58]:

(2) $$\eta_{\mathrm{ET}}=\left(1-\frac{\tau }{\tau_{0}}\right)\times 100\%$$

where τ and τ_0_ correspond to intrinsic the decay lifetime of the ^5^D_4_ excited level of Tb^3+^ in the presence and absence of acceptor ions (Eu^3+^) in the host lattice, respectively. Indeed, the shortening of the donor’s lifetime when the acceptor coexists is a measure of its ability for energy transfer. The energy transfer efficiencies for prepared xerogels are depicted in Table 1. The calculated efficiencies of Tb^3+^ → Eu^3+^ energy transfer for both of co-doped xerogels are comparable and they are close to η_ET_ ≈ 16%. The comparability in η_ET_ values is a consequence of the chemical similarity of the nearest framework around rare-earths (Tb^3+^, Eu^3+^) in xerogels. Such relatively low efficiencies are correlated with the long interionic distances between Tb^3+^ and Eu^3+^ ions dispersed within silicate sol-gel host. 

For glass-ceramic samples the recorded decay curves are well-fitted to double-exponential functions, which can be expressed by following Equation:

(3) $$I\left(t\right)/I_{0}=A_{1}\times \exp\left(-t/\tau_{1}\right)+A_{2}\times \exp\left(-t/\tau_{2}\right),$$

where τ_1_ is the decay time of a short lifetime component, τ_2_ is the decay time of a long component, while the A_1_ and A_2_ parameters are amplitudes at t = 0. The double-exponential character of the decay curves clearly means that Tb^3+^ ions are distributed between two different surroundings in which decay processes take place with variable rates. The first of them is a silicate sol-gel network in which the luminescence lifetimes of the ^5^D_4_ state of Tb^3+^ ions are shorter (τ_1_ decay component) due to presence of high-vibrational energy modes in their local framework (numerous Q^3^ units of SiO_4_ tetrahedrons (~1050 cm^−1^) and residual Si–OH groups (>3000 cm^−1^)). The second surrounding is according to fluorides, i.e., YF_3_ and LaF_3_ nanocrystals. Due to their low-phonon energies close to ~358 (YF_3_ phase) and ~350 cm^−1^ (LaF_3_ phase), the radiative relaxation from the ^5^D_4_ excited state is strongly promoted, and thus, the luminescence lifetimes are considerably prolonged (τ_2_ decay component). In other words, the change in decay profiles from mono-exponential functions (for xerogels) into double-exponential functions (for GCs) is evident proof, which allowed us to conclude that dopant ions are located either in a silicate sol-gel host or fluoride nanocrystals. 

It is quite interesting that the τ_2_(^5^D_4_) component for SiO_2_-LaF_3_:Tb^3+^ GC is longer (6.96 ± 0.27 ms) compared with the τ_2_(^5^D_4_) component for SiO_2_-YF_3_:Tb^3+^ GC (4.85 ± 0.40 ms). Since the phonon energies of YF_3_ and LaF_3_ fluoride phases are almost the same, we assumed that such difference in τ_2_ lifetime values may suggest that the migration of Tb^3+^ ions from silicate sol-gel host into LaF_3_ phase could be more efficient than into YF_3_ nanocrystals. It should be also pointed out that co-doping with Eu^3+^ ions lead to the considerable shortening of τ_2_(^5^D_4_) components due to occurrence of Tb^3+^ → Eu^3+^ ET process (3.10 ± 0.24 ms for SiO_2_-YF_3_:Tb^3+^,Eu^3+^ GC and 2.97 ± 0.26 ms for SiO_2_-LaF_3_:Tb^3+^,Eu^3+^ GC). To estimate the energy transfer efficiencies, we used the average luminescence lifetimes of the ^5^D_4_ state for glass-ceramic materials singly doped with Tb^3+^ and co-doped with Tb^3+^/Eu^3+^ ions calculated from the following Equation [59]:

(4) $$\tau_{\mathrm{avg}}=\frac{A_{1}\tau_{1}^{2}+A_{2}\tau_{2}^{2}}{A_{1}\tau_{1}+A_{2}\tau_{2}}\;$$

where A_1_ and A_2_ are fitting constants, and τ_1_ and τ_2_ are short and long decay components, respectively.

To determine the percentage contribution of short- (τ_1_) and long-lived (τ_2_) components involved in the total decay process, the following equations were used:

(5) $$\%,\tau_{1}=\frac{A_{1}}{A_{1}+A_{2}}\times 100\%,\;$$

(6) $$\%,\tau_{2}=\frac{A_{2}}{A_{1}+A_{2}}\times 100\%.$$

For both SiO_2_-YF_3_:Tb^3+^ as well as SiO_2_-YF_3_:Tb^3+^,Eu^3+^ GCs, the percentage contributions of τ_1_ and τ_2_ are quite comparable and equal nearly 50%. A slightly different observation was attained for GCs containing LaF_3_ nanocrystals. The difference in contribution of τ_1_ and τ_2_ decay components is slightly more visible for GCs containing LaF_3_ nanocrystals. For the sample singly doped with Tb^3+^ ions, the contribution of the τ_2_ component is slightly larger (56.42%) than that of the τ_1_ component (43.58%); meanwhile, co-doping with Eu^3+^ ions results in an increase in the τ_1_ component’s contribution (57.16%) and slight decrease in τ_2_ contribution (42.84%). The luminescence lifetimes of the ^5^D_4_ state of Tb^3+^ ions, their percentage contribution (%, τ_n_(^5^D_4_)), average lifetimes (τ_avg_) and the Tb^3+^ → Eu^3+^ ET efficiencies (η_ET_) for fabricated GCs are depicted in Table 2.

It was observed that the average luminescence lifetime for SiO_2_-LaF_3_:Tb^3+^,Eu^3+^ GC (τ_avg_(^5^D_4_) = 2.44 ms) is 2.6-fold shorter than for SiO_2_-LaF_3_:Tb^3+^ GC (τ_avg_(^5^D_4_) = 6.23 ms), while for SiO_2_-YF_3_:Tb^3+^,Eu^3+^ GC (τ_avg_(^5^D_4_) = 2.62 ms), it is 1.6-fold shorter than for SiO_2_-YF_3_:Tb^3+^ GC (τ_avg_(^5^D_4_) = 4.18 ms). The greater shortening of τ_avg_(^5^D_4_) luminescence lifetime for SiO_2_-LaF_3_:Tb^3+^,Eu^3+^ GC than for SiO_2_-YF_3_:Tb^3+^,Eu^3+^ GC indicates a more efficient Tb^3+^ → Eu^3+^ energy transfer. Indeed, the calculated ET efficiency value for SiO_2_-LaF_3_:Tb^3+^,Eu^3+^ (η_ET_ = 60.8%) is about 1.6-fold higher than for SiO_2_-YF_3_:Tb^3+^,Eu^3+^ (η_ET_ = 37.3%). It should also be noted that the transformation from xerogels into glass-ceramic materials during controlled heat-treatment strongly determines the Tb^3+^ → Eu^3+^ energy transfer efficiency, η_ET_, which increased from ~16% (xerogels) up to 37.3% (SiO_2_-YF_3_:Tb^3+^,Eu^3+^ GCs) and 60.8% (SiO_2_-LaF_3_:Tb^3+^,Eu^3+^ GCs).

**Figure 11:**
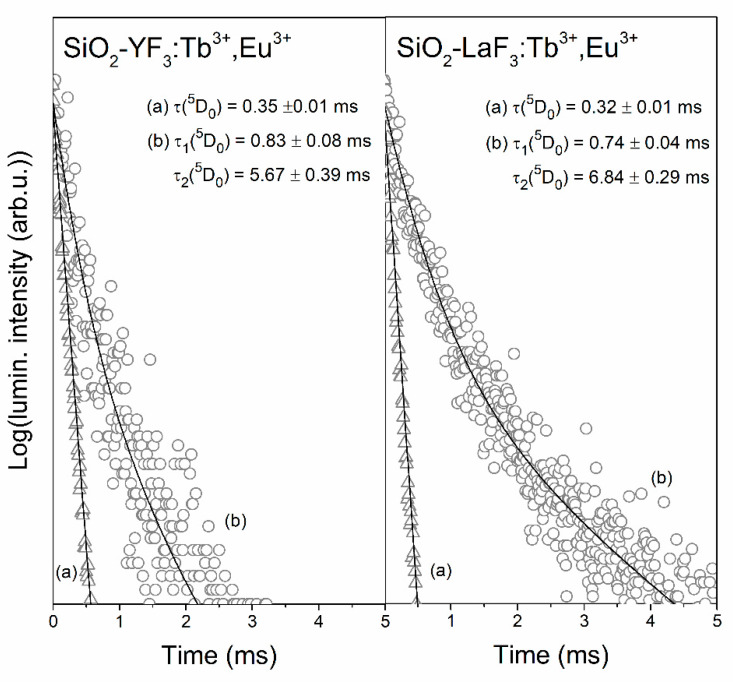
Luminescence decay curves of the ^5^D_0_ state of Eu^3+^ recorded for xerogels (**a**) and glass-ceramics (**b**) co-doped with Tb^3+^/Eu^3+^ ions.

The luminescence decay curves of the ^5^D_0_ state of Eu^3+^ ions in xerogels and SiO_2_-YF_3_:Tb^3+^,Eu^3+^ as well as SiO_2_-LaF_3_: Tb^3+^,Eu^3+^ glass-ceramics were recorded for λ_em_ = 611 nm (Figure 11). Similarly, as in the case of decay curves recorded for the ^5^D_4_ state of Tb^3+^ ions, the curves of the ^5^D_0_ level are well-fitted to mono-exponential functions for xerogels and to double-exponential functions for GCs. Relatively short luminescence lifetimes for xerogels are caused by the coordination of Eu^3+^ ions by high-vibrational OH groups, which are involved in the non-radiative depopulation of the ^5^D_0_ level (only four phonons are needed to cover energy gap, ∆E = 12,500 cm^−1^). Since controlled heat-treatment of xerogels resulted in the partial migration of Eu^3+^ ions inside fluoride nanocrystals, we distinguished two significantly different luminescence lifetimes (τ_1_, τ_2_). From the decays, it could be concluded that there is a clear correlation between the designated luminescence lifetimes and energy transfer efficiencies (η_ET_) related to the relative distribution of rare-earths between silicate sol-gel host and fluoride environment. The higher energy transfer efficiency for SiO_2_-LaF_3_:Tb^3+^,Eu^3+^ (η_ET_ = 60.8%) results in longer decay components (τ_1_(^5^D_0_) = 0.74 ± 0.04 ms, τ_2_(^5^D_0_) = 6.84 ± 0.29 ms) than for SiO_2_-YF_3_:Tb^3+^,Eu^3+^ (τ_1_(^5^D_0_) = 0.83 ± 0.08 ms, τ_2_(^5^D_0_) = 5.67 ± 0.39 ms) with lower Tb^3+^ → Eu^3+^ energy transfer efficiency (η_ET_ = 37.3%). Indeed, it was observed that the average luminescence lifetime for SiO_2_-LaF_3_:Tb^3+^,Eu^3+^ (τ_avg_(^5^D_0_) = 5.87 ms) is longer than the average lifetime for SiO_2_-YF_3_:Tb^3+^,Eu^3+^ (τ_avg_(^5^D_0_) = 4.59 ms). Such an effect could also indicate that the interionic distance between neighboring Tb^3+^ and Eu^3+^ ions in LaF_3_ nanocrystals seems to be shorter than in the YF_3_ crystal lattice. In other words, this could suggest that the segregation of rare-earths inside the LaF_3_ crystal phase is greater, which promotes the Tb^3+^ → Eu^3+^ ET. The luminescence decay times, their percentage distribution as well as average lifetimes for both types of fabricated GC sample are depicted in Table 3. 

The optical characterization of fabricated co-doped sol-gel samples was supplemented with calculations of quantum yields, Ф_Eu_. The quantum efficiencies were calculated from Ф = k_R_/k formula in which k is the total decay rate constant (k = 1/τ) and k_R_ is the radiative rate constant estimated from the following Equation [60]:

(7) $$k_{R}=A_{\mathrm{MD},0}n^{3}\left(\frac{I_{\mathrm{tot}}}{I_{\mathrm{MD}}}\right).$$

In above equation, A_MD,0_ is according to the Einstein spontaneous emission coefficient for the ^5^D_0_ → ^7^F_1_ transition (14.65 s^−1^ [61]); I_tot_ is the sum of integrated intensities of the ^5^D_0_ → ^7^F_J_ (J = 0–4) luminescence bands of Eu^3+^; I_MD_ is the integrated intensity of the ^5^D_0_ → ^7^F_1_ magnetic-dipole transition and n is the refractive index. The refractive index of YF_3_ and LaF_3_ nanocrystals is almost the same and equals n = 1.55–1.56. Such values are comparable with previously published results [62,63]. It is quite interesting that the refractive index of LaF_3_ nanocrystals is slightly lower than that of the corresponding LaF_3_ single crystal, as was proven by Z. Wang et al. [64]. Finally, the calculated quantum yields are depicted in Table 4. 

The quantum efficiencies for xerogels are comparable and equal to 9.2% for XG-Y/Tb^3+^,Eu^3+^ and 8.1% for XG-La/Tb^3+^,Eu^3+^. Much greater differences in quantum efficiencies were denoted for fabricated glass-ceramics. In fact, the Ф_Eu_ calculated for SiO_2_-YF_3_:Tb^3+^,Eu^3+^ GC equal to 49.5%, meanwhile for SiO_2_-LaF_3_:Tb^3+^,Eu^3+^ GC sample, the Ф_Eu_ value achieve even 73.0%. The observed significant difference is clearly related to Tb^3+^ → Eu^3+^ energy transfer, the efficiency of which is much greater for SiO_2_-LaF_3_:Tb^3+^,Eu^3+^ (η_ET_ = 60.8%) compared with SiO_2_-YF_3_:Tb^3+^,Eu^3+^ glass-ceramics (η_ET_ = 37.3%). Based on the current literature, the luminescence quantum yields for Tb^3+^,Eu^3+^ co-doped nanocrystals are usually in a range from 32% to 61% [65]. It is very interesting that the Ф_Eu_ value for SiO_2_-YF_3_:Tb^3+^,Eu^3+^ is in good accordance with the presented data, but the SiO_2_-LaF_3_:Tb^3+^,Eu^3+^ GC sample is characterized by a greater quantum efficiency value. Furthermore, as was proven by N. Shrivastava et al. [66], the quantum yields for LaF_3_:Eu^3+^ nanocrystals could vary from 67% up to 85% depending on the Eu^3+^ concentration. Therefore, the obtained results suggest that fabricated sol-gel glass-ceramics could be considered as quite good candidates for visible light-emitting devices.

## 4. Conclusions

In this work, SiO_2_-MF_3_:Tb^3+^,Eu^3+^ (M = Y, La) glass-ceramic materials were prepared via sol-gel method during the controlled heat-treatment of silicate xerogels at 350 °C. The performed systematic photoluminescence measurements confirmed that fabricated sol-gel materials exhibit multicolor emission due to the coexistence of the characteristic emission bands originating from both dopant ions, i.e., Tb^3+^ (^5^D_4_ → ^7^F_J_, J = 6–3) and Eu^3+^ (^5^D_0_ → ^7^F_J_, J = 0–4) due to Tb^3+^ → Eu^3+^ energy transfer occurrence. Based on performed luminescence decay analysis from the ^5^D_4_ (Tb^3+^) and the ^5^D_0_ (Eu^3+^) excited levels, the correlation between luminescence lifetimes and the distribution of rare-earth ions between the silicate sol-gel host and fluoride nanocrystals was clearly proven. Hence, it was suggested that more preferable segregation of rare-earth ions inside LaF_3_ nanocrystals occurred. Moreover, the transformation from xerogels into glass-ceramic materials during controlled heat-treatment determines the strong influence on Tb^3+^ → Eu^3+^ energy transfer efficiency, η_ET_, which increased from ~16% (xerogels) up to 37.3% and 60.8% (SiO_2_-YF_3_:Tb^3+^,Eu^3+^ and SiO_2_-LaF_3_:Tb^3+^,Eu^3+^ glass-ceramics, respectively). The obtained luminescent results clearly suggest that the prepared sol-gel glass-ceramic materials could be considered as promising candidates for use as optical elements in RGB-lighting optoelectronic devices operating under NUV excitation.

## Figures and Tables

**Table 1:** Luminescence lifetimes of the ^5^D_4_ state of Tb^3+^ (τ) and ET efficiencies (η_ET_) for fabricated silicate xerogels.

Sample	τ(^5^D_4_) (ms)	η_ET_ (%)
XG-Y/Tb^3+^,Eu^3+^	0.82 ± 0.03	15.5
XG-Y/Tb^3+^	0.97 ± 0.01	-
XG-La/Tb^3+^,Eu^3+^	0.75 ± 0.03	15.7
XG-La/Tb^3+^	0.89 ± 0.02	-

**Table 2:** Luminescence lifetimes of the ^5^D_4_ state of Tb^3+^, their contribution and energy transfer (ET) efficiencies (η_ET_) for fabricated glass-ceramics.

Sample	τ(^5^D_4_) (ms)	%,τ_n_(^5^D_4_)	τ_avg_(^5^D_4_) (ms)	η_ET_ (%)
SiO_2_-YF_3_:Tb^3+^,Eu^3+^	0.86 ± 0.10 (τ_1_) 3.10 ± 0.24 (τ_2_)	49.79% (τ_1_) 50.21% (τ_2_)	2.62	37.3
SiO_2_-YF_3_:Tb^3+^	0.94 ± 0.12 (τ_1_) 4.85 ± 0.40 (τ_2_)	51.50% (τ_1_) 48.50% (τ_2_)	4.18	-
SiO_2_-LaF_3_:Tb^3+^,Eu^3+^	0.67 ± 0.07 (τ_1_) 2.97 ± 0.26 (τ_2_)	57.16% (τ_1_) 42.84% (τ_2_)	2.44	60.8
SiO_2_-LaF_3_:Tb^3+^	1.34 ± 0.12 (τ_1_) 6.96 ± 0.27 (τ_2_)	43.58% (τ_1_) 56.42% (τ_2_)	6.23	-

**Table 3:** Luminescence lifetimes of the ^5^D_0_ state of Eu^3+^ and their contributions for fabricated glass-ceramics.

Sample	τ(^5^D_0_) (ms)	%, τ_n_(^5^D_0_)	τ_avg_(^5^D_0_) (ms)
SiO_2_-YF_3_:Tb^3+^,Eu^3+^	0.83 ± 0.08 (τ_1_) 5.67 ± 0.39 (τ_2_)	66.30% (τ_1_) 33.70% (τ_2_)	4.59
SiO_2_-LaF_3_:Tb^3+^,Eu^3+^	0.74 ± 0.04 (τ_1_) 6.84 ± 0.29 (τ_2_)	63.58% (τ_1_) 36.42% (τ_2_)	5.87

**Table 4:** Quantum yields (Ф_Eu_) calculated for fabricated xerogels and glass-ceramic materials.

Sample	Ф_Eu_ (%)
XG-Y/Tb^3+^,Eu^3+^	9.2
XG-La/Tb^3+^,Eu^3+^	8.1
SiO_2_-YF_3_:Tb^3+^,Eu^3+^	73.0
SiO_2_-LaF_3_:Tb^3+^,Eu^3+^	49.5
